# The Role of JMY in p53 Regulation

**DOI:** 10.3390/cancers10060173

**Published:** 2018-05-31

**Authors:** Omanma Adighibe, Francesco Pezzella

**Affiliations:** Nuffield Division of Clinical Laboratory Science—Radcliffe Department of Medicine, University of Oxford, John Radcliffe Hospital, Oxford OX3 DU, UK

**Keywords:** p53, JMY, regulation, apoptosis, motility

## Abstract

Following the event of DNA damage, the level of tumour suppressor protein p53 increases inducing either cell cycle arrest or apoptosis. Junctional Mediating and Regulating Y protein (JMY) is a transcription co-factor involved in p53 regulation. In event of DNA damage, JMY levels also upregulate in the nucleus where JMY forms a co-activator complex with p300/CREB-binding protein (p300/CBP), Apoptosis-stimulating protein of p53 (ASPP) and Stress responsive activator of p53 (Strap). This co-activator complex then binds to and increases the ability of p53 to induce transcription of proteins triggering apoptosis but not cell cycle arrest. This then suggests that the increase of JMY levels due to DNA damage putatively “directs” p53 activity toward triggering apoptosis. JMY expression is also linked to increased cell motility as it: (1) downregulates the expression of adhesion molecules of the Cadherin family and (2) induces actin nucleation, making cells less adhesive and more mobile, favouring metastasis. All these characteristics taken together imply that JMY possesses both tumour suppressive and tumour metastasis promoting capabilities.

## 1. Introduction

**Figure 1:**
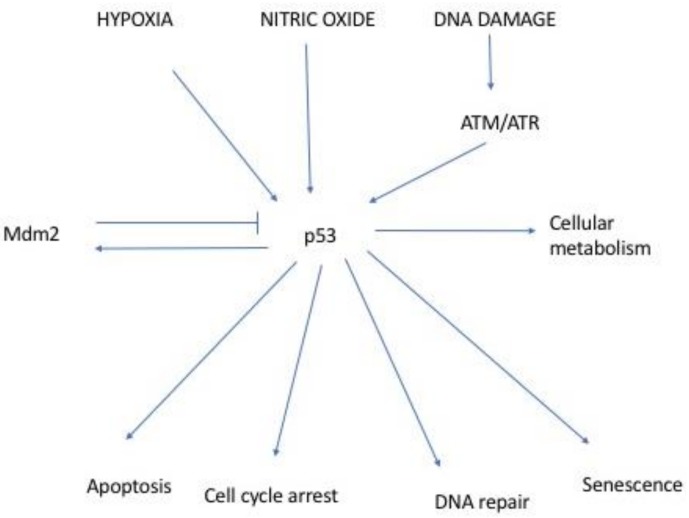
Basic functions of p53. p53 levels are tightly controlled and in unperturbed cells MDM2 is its main regulator via induction of p53 degradation. There is a negative feedback loop between the two proteins. p53 itself induces MDM2 transcription, hence as p53 levels increase, more MDM2 is produced, which in turn down regulates p53 levels. In the presence of DNA damage and other stresses, p53 degradation stops and its levels increase. Elevated levels of stabilized p53 induce transcription of proteins involved in different types of responses, particularly cell cycle arrest and apoptosis. Modified schematic diagram [8].

A salient observation in cancer biology has been that TP53 is frequently mutated in many human tumours [1,2]. p53 protein was identified in SV40 transformed cells where it was associated with Large T Antigen. It was later discovered to be a prominent transcription factor whose function is essential in preventing inappropriate cell proliferation and maintaining genome integrity following genotoxic stress (Figure 1) [2,3]. Consequently, in response to cellular stress such as DNA damage, hypoxia, oncogene overexpression and viral infection, the p53 protein expression level is augmented. Post translational modifications, which include phosphorylation, acetylation, ubiquitination, and methylation, stabilize p53 enabling it to activate multiple promoter elements of target genes affecting cellular processes such as cell cycle arrest, senescence, and apoptosis [2,4].

p53 regulation of apoptosis (programed cell death) is at the heart of neoplastic proliferative control. In mammalian cells, apoptosis can be either p53 transcriptional dependent or p53 transcriptional independent. The p53 transcriptional dependent mechanism is set in motion by cellular stress like DNA damage which triggers p53 post translational modification of phosphorylation and acetylation stabilizing p53. Stabilization enables p53 to bind and activate pro-apoptotic genes. It is highly speculated that p53 selectively activates transcription of pro-apoptotic target genes upon interaction with transcriptional co-activators such as p300/CREB-binding protein (p300/CBP), Junctional and regulatory protein (JMY), Stress responsive activator of p53 (Strap) and Apoptosis-stimulating protein of p53 (ASPP) [2,5].

Some studies have shown that inhibition of mRNA and protein synthesis which inhibit transcription of p53 target genes did not inhibit p53 dependent apoptosis. A plausible explanation for this is the existence of an alternative p53 dependent apoptosis that is transcriptionally independent. According to Bossi et al., studies corroborating this hypothesis show that upon DNA damage, p53 can localize to the mitochondria where it triggers a rapid apoptotic response which occurs even before p53 target genes are activated in the nucleus. This alternate apoptotic response is attained by the p53 DNA binding domain directly binding pro-apoptotic proteins BCL-XL and BCL2 within the mitochondria facilitating the release of BH3 protein and induction of mitochondrial permeabilization and apoptosis [2,6].

The activity of p53 is tightly controlled and regulated at multiple levels and the importance of co-factors influencing these processes is becoming increasingly evident [5]. Mouse Double Minute 2 homolog (MDM2) for instance is a major negative regulator of p53 (Figure 1). MDM2’s regulatory capacity can be by direct p53 ubiquitination targeting p53 protein for degradation, or indirectly via p53 cofactors ubiquitination also marking them for degradation [5,7]. Ubiquitination is accomplished through MDM2’s E3 ubiquitin ligase activity, which promotes proteasome-dependent degradation and modulates nuclear export [2].

CBP and p300 are a family of acetyltransferase which act as transcriptional activators for several transcription factors including p53 [9]. Following DNA damage, p53 is phosphorylated at Serine15 stabilizing and enabling it to bind p300. Upon ligation with p300, two sites on the C terminal region of p53 are acetylated. It has been proposed that following acetylation, p53’s stability and recruitment of its targets are further enhanced by the presence of these co-activators leading to a preponderant transcription of p53 target proteins [9].

The Junctional Mediating and regulator Y protein (JMY) was originally identified as a CBP/p300 co-factor regulating p53 activity [10]. Upon DNA damage, it was observed that JMY interacts with and forms a complex with p300 and Strap while recruiting Protein Arginine Methyltransferase 5 (PRMT5) into a co-activator complex, triggering p53 response [11]. According to Coutts et al., JMY’s functional role in p53 response is evident in its associated increase with p53-dependent transcription induced apoptosis. The transcriptional co-factor role of JMY is observed where its increased expression translates to increased transcription of factors downstream to p53 without altering p53 protein levels [9]. Studies have also shown JMY to be an MDM2 target for ubiquitination and degradation as one of the mechanisms via which MDM2 regulates p53 activity. This also supports the fact that upon DNA damage, JMY is released from MDM2, making it available to contribute to p53’s response to DNA damage [9].

Metastasis is facilitated by increased cell motility, allowing tumour cells to invade and colonize surrounding as well as distant tissues. In addition to its role in p53 induced apoptosis, JMY also participates in the enhancement of cell motility [11,12]. JMY contains a series of WASP-Homology 2 (WH2) domains that promote actin nucleation or elongation enabling it to promote cell motility [11]. Studies have shown JMY to nucleate actin in vitro and induce actin filament formation in vivo due its inherent WH2 domain series [11,13]. It is via its WH2 domain that JMY is also able to down-regulate E-cadherin, an adherent junction protein required for cell-cell adhesion which is known to be lost during the course of tumor progression [14,15]. This loss of E-cadherin also favors cell motility, metastasis, and invasion. 

This in effect supports the hypothesis, generated by micro array study showing an inverse relationship between JMY and Trap1 [16]: hypoxia leads to slower proliferation with decreased Trap1 and increased JMY, which in turn promotes cell motility facilitating escape from the hypoxic environment. 

Interesting, Coutts et al. have demonstrated that JMY localizes in the nucleus and cytoplasm but following stress or DNA damage, JMY migrates from the cytoplasm to the nucleus to transcriptionally enhance P53’s response [11]. According to this group, when JMY shuttles into the nucleus, JMY’s contribution to cell motility diminishes [11]. This then implies that JMY is playing a dichotomous role in cancer biology: (1) having a tumour suppressive capacity in the event of DNA damage where it enhances p53 activity and (2) functioning as a putative tumour metastasis promoter due to its ability to downregulate E-cadherin, nucleate actin filament and contribute to cell motility. It is then important to reconcile the role and cue of JMY in these two different cellular processes of programmed cell death versus actin dynamics regulation of tumourigenesis. 

## 2. Identification of JMY

JMY was originally identified by Shikama et al. [10] while using the two-hybrid method to screen for proteins participating in the p300/CPB proteins complex and involved in the regulation of p53 transcription [10]. JMY was found to be a protein of 110 kDa whose gene is located on chromosome 5 at the 5q 13.2 band. Zuchero et al. demonstrated in HL60 cells that JMY primarily localizes in the nucleus but can move between the nucleus and cytoplasm [13]. DNA damage is one of the triggers for activation of factors that regulate JMY such as: (1) E2F1 which mediates increased expression of JMY; (2) Strap and p300 which form a co-activator complex with JMY switching on p53 induced apoptosis; and (3) MDM2 which releases bound JMY to participate in p53 induced apoptosis response. 

### 2.1. JMY Regulation by E2F1

**Figure 2:**
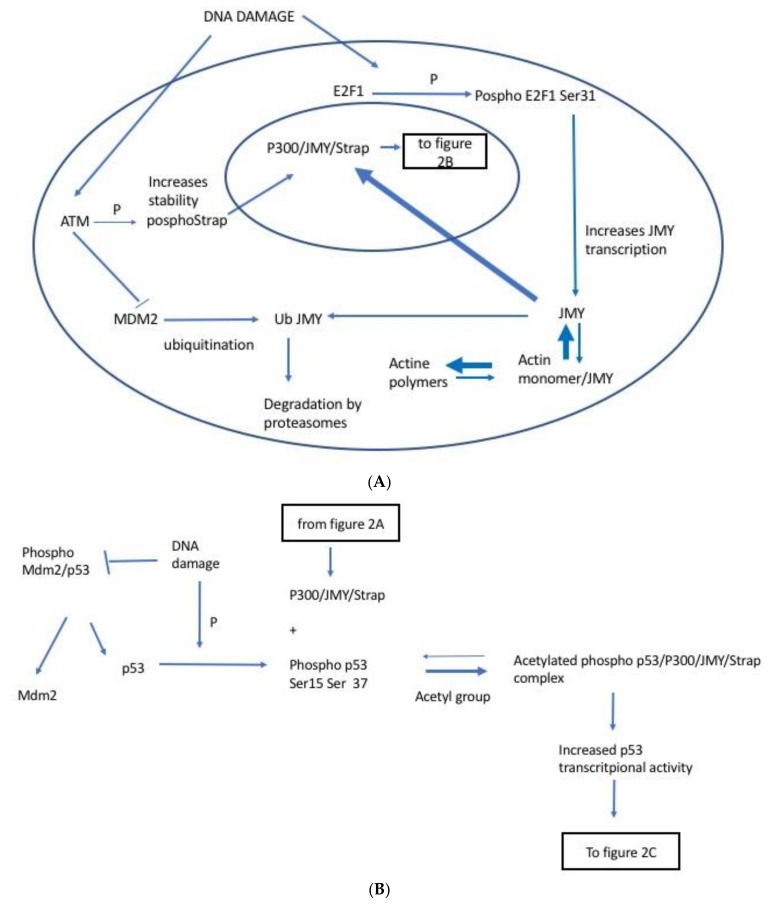

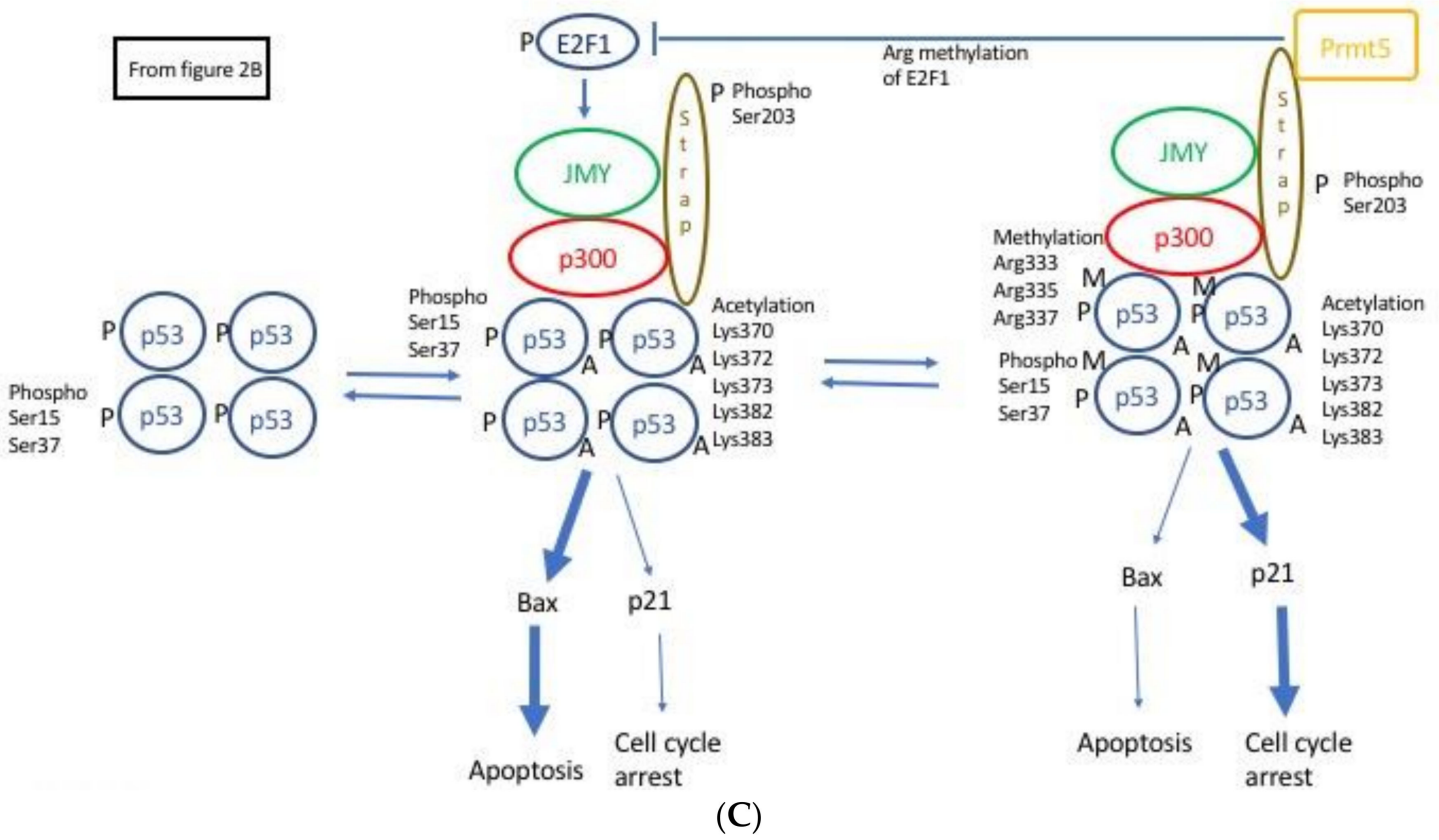
JMY (Junctional Mediating and Regulating Y protein)/P53 interaction pathway. (**A**) JMY’s regulation. In unperturbed cells, JMY levels are maintained at a constant state by a balance between transcription and degradation. The latter is controlled by Mdm2 which ubiquitinates JMY leading to its degradation by proteasomes. Following DNA damage, the newly phosphorylated E2F1 induces increased transcription of JMY, while ataxia-telangiectasia mutated (ATM) dampens MDM2 activity. Furthermore, actin monomers form polymers and therefore are no longer available to bind to JMY and sequester it in the cytoplasm. Since it is no longer bound to actin monomers, JMY can then bind to Importin and translocate to the nucleus. This causes the levels of JMY in the nucleus to increase and form a complex with P300. Stability of this complex is further increased by linkage with phosphorylated Stress responsive activator of p53 (Strap). Based on: [18,19,20,21,22]. (**B**) JMY’s effect on p53. Following DNA damage, p53 is phosphorylated and escapes degradation resulting in upregulation of p53 levels. Phosphorylated p53 binds to the JMY/p300/Strap complex and its transcriptional activity increases. Based on: [9]. (**C**) Formation of molecular complexes and their effects on apoptosis and cell cycle. Following DNA damage, p53 is phosphorylated and released from MDM2. It binds to the p300/JMY/Strap complex which causes acetylation of five Lysine residues located on the C terminus region of p53. This leads to an increased ability of p53 to transcribe Bax, but not p21, resulting in a preferential activation of the apoptotic pathway over cell cycle arrest. However, when PRMT5 is recruited to Strap that is bound to the JMY/p300 complex, this triggers the methylation of p53 shifting the process away from Bax transcription and apoptosis to increased transcription of p21 and induction of cell cycle arrest. PRMT5 further reinforces this switch by E2F1 inhibition via methylation which reduces JMY’s transcription. Based on and modified from: [9,23,24,25,26,27].

Transcription of the E2F family of proteins can lead to either cell proliferation or apoptosis, hence suggesting a dichotomy in their function [17]. DNA damage activates Retinoblastoma (Rb) to bind E2F and block transcription [17]. In tandem E2F1, the most potent apoptotic inducer of the E2F family (Figure 2A), is activated causing transcription at the pro-apoptotic promoters and effecting apoptosis. There are two main forms of E2F1 residual phosphorylation and both induce apoptosis in the event of DNA damage. Serine 364 phosphorylated E2F1 binds to Rb (Rb-E2F1), while Serine 31 phosphorylated E2F1 does not bind Rb and remains free. Both the E2F1-Rb complex and the free E2F1 are essential for maximal induction of apoptosis [17]. Levels of JMY protein increase in cells treated “in vitro” with DNA damaging compounds like ultraviolet light, etoposide, and actinomycin D [5]. JMY transcription and JMY protein accumulation are induced by the transcription of E2F1 activated by DNA damage [18]. According to Carnevale et al., DNA damage signals activate both pRB and E2F1 to help activate apoptosis [17]. Some studies have shown that active E2F1 introduced into the human cell line U2OS induces transcription of JMY and three others pro-apoptotic p53 co-factors: Aspp1, Aspp2, and TP53Inp1 [18]. Also, inhibition of protein synthesis has been shown not to prevent the increased transcriptions of these factor in the presence of E2F1 which suggest that these genes are directly targeted by E2F1. Interestingly, while Aspp1, Aspp2, and TP53Inp1 have putative E2F1 binding sites, no such region has been found on JMY. However, this finding does not exclude the possibility of interaction between E2F1 and JMY [18].

### 2.2. JMY Regulation by Strap

Strap is another co-factor involved in the p53 regulation through its interaction with both p300/JMY in a co-activation complex. Strap contains six tetratricopeptide (TPR) repeat motifs composed of several protein binding regions which can allow Strap protein to bind different substrates and form multiple complexes [9,20,21]. Following DNA damage, a protein kinase ATM phosphorylates Strap on Serine 203 (Figure 2A). Phosphorylated Strap stabilizes and accumulates in the nucleus where it binds to the CBP/p300/JMY complex. Strap in fact upregulates JMY levels and strengthens its interaction with p300. Co-immunoprecipitation studies have shown that there is an increased level of JMY in the p300 immunocomplex in the presence of Strap. This is likely due to the influence of Strap and its TPR effect on the recruitment of JMY to the p300 co-activator [20]. Using a two-hybrid assay and challenging free p300 and JMY with added Strap, it was demonstrated that the presence of Strap increased the number of p300/JMY complexes. Ultimately, the stabilization of the p300/JMY complex leads to increased p53 transcriptional activity (Figure 2B) [9,20,21].

### 2.3. JMY Regulation by MDM2

MDM2 ubiquitinates p53 causing its degradation. However, in the event of DNA damage when p53 is phosphorylated, there is minimal interaction between p53 and MDM2 allowing its escape from degradation (Figure 2B) [5,9]. Levels of JMY protein are also subject to regulation by MDM2 in stress-free cells. JMY and MDM2 physically interact through the C-terminal domain of MDM2 which harbours the E3 ligase [5]. Coutts et al. have shown that when stress free mouse embryonic fibroblasts are treated with inhibitors of MDM2 ligase, levels of JMY protein increase. This finding suggests that in absence of stress, JMY protein production and degradation is maintained in a steady state by regulation mediated via MDM2. Following induction of DNA double strand break by ultraviolet radiation or by actinomycin-D, JMY levels increase, not only because of increased transcription, but also because of diminished degradation by MDM2 [5,7]. With the minimal JMY inhibition by MDM2, JMY, Strap, and p300 complex induced acetylation of p53 which also contributes to its protection from degradation by MDM2 [7] 

### 2.4. JMY Increases p53 Dependent Transcription Leading to Selective Increase in Apoptosis

Higher levels of p300/JMY increase p53 transcriptional activity leading to increased apoptosis but not cell cycle arrest [10]. As discussed above, this co-activator complex’s activity is further enhanced in the presence of Strap. p53 downstream genes such as Bax- which leads to apoptosis- and p21- which induces cell cycle arrest; have been investigated in regards to their association with p300/JMY complex. Levels of Bax were observed to increase in the presence of p300/JMY while those of p21 were very modest (Figure 2C) [10]. This result substantiates the hypothesis that when activated, the p300/JMY co-activator complex in association with p53 induces the pro-apoptotic pathway with upregulation of apoptotic proteins like Bax. 

P53 activation can lead to both cell cycle arrest and apoptosis, or just one of the two processes. However, the cue for p53 to activate just one of either processes/pathways had remained unknown. As recently unveiled, the path to apoptosis is via JMY and activation of other pro-apoptotic factors [18]. In the event of DNA damage, phosphorylated p53 binds to the p300/JMY/Strap complex which causes acetylation of five Lysine residues located on the C terminus region of p53. This activation increases the p53 transcription of Bax, but not p21, resulting in a preferential activation of the apoptotic pathway without cell cycle arrest. However, if PRMT5 is recruited to Strap bound to the JMY/p300 complex, this triggers the methylation of p53, shifting the process away from Bax transcription and apoptosis to increased transcription of p21 and induction of cell cycle arrest. This in turn causes the downregulation of JMY that further orients that process away from the pro-apoptotic pathway (Figure 2C) [23,24].

### 2.5. JMY and Cell Motility

Another prominent role of JMY is to regulate motility by affecting actin nucleation and cell adhesion [11]. Actin filaments provide the structural basis for cell motility and are critical to numerous physiological processes such as morphogenesis, wound healing, migration, membrane transport, and metastasis [28]. Actin filament formation occurs either via branching of already existing actin filament or alternatively via de-novo nucleation of actin monomers [28]. 

Spontaneous assembly and nucleation of actin trimers and dimers are kinetically unfavourable. To counteract this obstacle, cells use actin nucleators and nucleation-promoting factors (NPF) to jump start actin nucleation and filament formation. Actin filament formation via branching is usually facilitated by the actin nucleator “Actin related protein 2/3 complex” (Arp2/3). Arp2/3 is activated by NPFs via their ‘Wiskot-Aldrich Syndrome protein (WASp) homology-2’domain (also known as WH2 domain), which are actin binding motifs that enable assembly of actin monomers [29]. Alternatively, the de-novo nucleation is produced by nucleators such as ‘SPIRE which themselves contain WH2 domains and do not require activation by NPFs [13,29].

With the discovery of JMY protein sequence homology to actin regulators and nucleators, came the knowledge of its involvement with actin [13]. JMY’s capacity to regulate actin dynamics relies on its possession of WH2 domains which can either independently initiate actin filament formation in SPIRE-like fashion or activate actin nucleators such as Arp2/3 [29]. Experiments with myeloid lineage HL60 have shown that JMY is mostly nuclear in these cells but when differentiation into neutrophil is induced some JMY protein move to the cell edge where they co-localize with actin. JMY overexpression was also found to be associated with increased speed for migrating cells [13]. Additionally, hypoxia inducible factor (HIF) stimulation, synonymous with a tumourigenic environment, causes increased JMY expression and cytoplasmic co-localization with actin [30]. Since actin filament nucleation facilitates cell motility and migration, JMY’s co-localization with it suggests a significant role in cell motility for JMY. 

**Figure 3:**
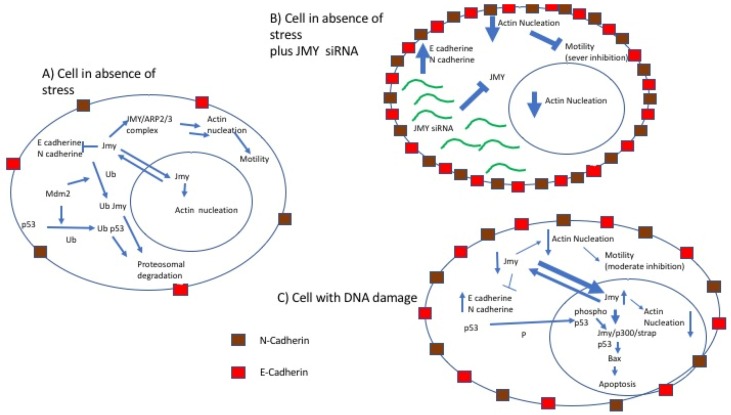
Model of JMY-mediated co-ordination between cell motility and DNA damage response in cell lines. A model of how JMY links p53 response to DNA damage and cell motility in cell lines. (**A**) In a motile cell, in absence of stress, the amount of JMY in the nucleus and in the cytoplasm are maintained in equilibrium. The available JMY protein in the cytoplasm inhibits E and N cadherin adhesion molecules in a dose dependent fashion and induces actin nucleation for cell motility both in an Arp2/3 dependent and independent fashion. (**B**) If such a cell is treated with siRNA targeting JMY, the inhibition of JMY transcription leads to a strong upregulation of the E and N cadherins and a reduction of actin nucleation, attenuating cell motility. (**C**) When DNA damage occurs, a more drastic drop in motility is observed. The explanation for this drastic drop in motility is the translocation of JMY from the cytoplasm to the nucleus diminishing the cytoplasmic JMY level. This however is in part compensated for by the overall increase of JMY levels following the DNA damage. This implies that JMY is capable of producing both forms of biochemical actin filaments. Having both forms of biochemical actin polymerization would also imply that JMY’s contribution to cell motility is via its nucleation filament and that JMY can promote rapid assembly of a new actin network by harnessing its ability to first nucleate new mother filaments and then activate Arp2/3 to branch off these filaments. This duality of JMY localization and functions in the cytoplasm and nucleus might be a characteristically and evolutionarily gained advantage [13,28].

Zuchero et al. [13] and Firat-Karalar et al. [31] demonstrated that purified JMY biochemically activates Arp2/3 (Figure 3) induced actin polymerization in a dose dependent fashion. JMY does not induce elongation of preformed filaments but is able to nucleate new filaments, speed up elongation and create barbed ends. These authors also demonstrated that due possession of WH2 domains, JMY is able to catalyze new filament formation in a SPIRE-like fashion, always in a dose-dependent fashion, even in the absence of Arp2/3 activity [13,28]. JMY’s nucleation activity in the presence of Arp2/3 complex occurs in the cytoplasm and leads to production of branched filaments. Once in the nucleus, JMY acts with a mechanism similar to that used by the actin nucleation factor SPIRE and produces unbranched filaments in an Arp2/3 independent fashion.

We previously discussed how JMY accumulates in the nucleus in response to DNA damage. However, another mechanism regulating its subcellular localization has been unveiled, this time in response to polymerization of actin monomers which ordinarily would bind to the JMY’s WH2 domains inhibiting JMY’s transfer to the nucleus [22]. This hypothesis is corroborated by studies which show JMY’s intranuclear localization after treatment with Jasplakinolide, a compound that induces polymerization of actin [32]. This intranuclear localization of JMY can be explained by the fact that since all the actin monomers are recruited to form polymers, no free monomers are available to bind to JMY. This further implies that JMY is no longer constrained in the cytoplasm and is then at liberty to translocate into the nucleus [32]. A similar observation was made when a mutation of JMY’s WH2 domain was introduced with the aim of preventing the binding of actin monomers [22]. 

The binding of an actin monomer to JMY can prevent JMY’s transfer to the nucleus, because it competes with Importin’s binding to JMY, since the Actin binding region overlaps with the Nuclear Localization Signal region targeted by Importin [22]. Importin binding to JMY has been demonstrated to be responsible for the transfer of JMY to the nucleus after DNA damage [22]. UV-induced DNA damage induces actin polymerization in the cytoplasm, just like treatment with Jasplakinolide [33]. Zuchero et al. have demonstrated a nuclear accumulation of JMY after exposure to UV or treatment with other DNA-damaging agents like Etoposide and Neocarzinostatin. Interestingly, JMY’s nuclear accumulation is conditional to the availability of Importin and unavailability of actin monomers. The authors concluded that the nuclear accumulation of JMY following DNA damage may be regulated by DNA damage induced actin assembly into polymers [22].

The second mechanism by which JMY affects motility is via its regulation of adhesion molecules E-Cadherin and N-Cadherin levels [11]. We have shown from work done in our laboratory that the expression of JMY is inversely associated with the levels of these two adhesion molecules. Additionally, our correlation studies of JMY and E Cadherin expression in 235 invasive breast carcinoma also show an inverse correlation between cytoplasmic JMY and membranous E cadherin [12]. Coutts et al. have also observed an upregulation of E-cadherin in JMY depleted MCF7 cells and the opposite effect is seen in JMY rich cells [11]. This then connotes a role for JMY in facilitating cell mobility via downregulation of adhesion molecules as the loss of E-cadherin also favors cell motility, metastasis and invasion [14,15].

## 3. Linking p53 Pathway and Cell Motility

It has become increasingly evident that JMY plays prominent roles in coordinating DNA damage response and cell motility. Considering JMY’s association with p53, this also implies that p53 plays a role in inhibition of cell motility (Figure 3) [34]. p53 has been found to be associated with tubulin, vimentin, F-actin, and tubulin indicating its possible role in cytoskeleton regulation. p53’s interaction with Cdc42 inhibits both filo podia formation, cellular polarization and “in vitro” cellular spreading. Furthermore, inhibition or absence of p53 leads to increased activity of the Rho pathway with consequent increase in cell migration [34].

JMY’s capacity to regulate cell motility and DNA damage response suggest that JMY could be the link between these two cellular processes. In the model, currently being explored (illustrated in Figure 3), following DNA damage, the net JMY concentration in the cytoplasm diminishes while its level in the nucleus increases as JMY translocates into the nucleus. In the nucleus most of the JMY is sequestrated by the Cp300/Strap/p53 complex to help stabilize p53. As a consequence, there is limited JMY protein available for actin nucleation and cadherin inhibition, which also implies a limited mobility for cells [11,22,35,36].

## 4. Conclusions

Having the ability to promote p53 induced apoptosis on one hand, and facilitate invasiveness of the cancer cells on the other, JMY is an example of genes that can act both as a suppressor gene and as a gene promoting tumour progression [37]. We have discussed here the role of JMY as a tumor suppressor, facilitating p53 induced apoptosis without cell cycle arrest in the event of DNA damage. While JMY’s putative tumour promoting role could be seen in its ability to increase cell motility and downregulate cadherin proteins. Potentially these unique and apparently contrasting roles of JMY can be reconciled with each other: In the event of DNA damage, the net JMY concentration diminishes in the cytoplasm and increases in the nucleus where most of the JMY is sequestrated by the Cp300/Strap/p53 complex. With a decreased amount of JMY available in the cytoplasm, there is less potential for continued actin nucleation and cell motility. It then seems that the cue to switch from pro metastatic role to tumor suppressive role may be the DNA damage signals. Further work however, is required to confirm this speculative hypothesis.
